# Transcriptome analysis based on a combination of sequencing platforms provides insights into leaf pigmentation in *Acer rubrum*

**DOI:** 10.1186/s12870-019-1850-7

**Published:** 2019-06-06

**Authors:** Zhu Chen, Xiaoyu Lu, Yun Xuan, Fei Tang, Jingjing Wang, Dan Shi, Songling Fu, Jie Ren

**Affiliations:** 1 Institute of Agricultural Engineering, Anhui Academy of Agricultural Sciences, Hefei, 230031 China; 2 College of Forestry and Landscape Architecture, Anhui Agricultural University, Hefei, 230036 Anhui China

**Keywords:** Leaf coloration, Molecular regulation, Anthocyanin, Chlorophyll, Carotenoid, *Acer rubrum*, Transcriptome analysis

## Abstract

**Background:**

Red maple (*Acer rubrum* L.) is one of the most common and widespread trees with colorful leaves. We found a mutant with red, yellow, and green leaf phenotypes in different branches, which provided ideal materials with the same genetic relationship, and little interference from the environment, for the study of complex metabolic networks that underly variations in the coloration of leaves. We applied a combination of NGS and SMRT sequencing to various red maple tissues.

**Results:**

A total of 125,448 unigenes were obtained, of which 46 and 69 were thought to be related to the synthesis of anthocyanins and carotenoids, respectively. In addition, 88 unigenes were presumed to be involved in the chlorophyll metabolic pathway. Based on a comprehensive analysis of the pigment gene expression network, the mechanisms of leaf color were investigated. The massive accumulation of Cy led to its higher content and proportion than other pigments, which caused the redness of leaves. Yellow coloration was the result of the complete decomposition of chlorophyll pigments, the unmasking of carotenoid pigments, and a slight accumulation of Cy.

**Conclusions:**

This study provides a systematic analysis of color variations in the red maple. Moreover, mass sequence data obtained by deep sequencing will provide references for the controlled breeding of red maple.

**Electronic supplementary material:**

The online version of this article (10.1186/s12870-019-1850-7) contains supplementary material, which is available to authorized users.

## Highlights

We applied a combination of next-generation sequencing (NGS) and single-molecule real-time sequencing (SMRT) to provide complete view of the ***Acer rubrum*** transcriptome, with further insights into color change in leaves.

## Background

The genus *Acer* (Aceraceae) is an essential decorative plant for landscapes due to its pleasing form and attractive autumn colors, which encompasses approximately 129 species with many infraspecific taxa [1]. The vast majority of these species are trees or shrubs that are primarily distributed across the temperate regions of East Asia, Eastern North America, and Europe [2]. As an important and unique member of the genus *Acer*, red maple (*Acer rubrum* L.) is endowed with high ornamental value, which is used more commonly in parks, urban spaces, and gardens.

The coloration and changes in colorful plants are very complex, where this quality trait is primarily determined by their metabolic composition. As one of the major bioactive constituents of *A. rubrum*, anthocyanins have garnered much attention due to their considerable potential to control leaf color changes in the spring and autumn [3]. Cyanidin-3-(2′, 3′-digalloyl-β-glucopyranoside) was the first known case of an anthocyanin diacylated with gallic acid [4], which was isolated from the red leaves of *A. platanoides*. To date, eight chemical compounds associated with the synthesis and accumulation of anthocyanin have been identified in *Acer* plants [4–10]; however, related substances were not discovered in *A. rubrum* until recently. Anthocyanins, which comprise a class of flavonoid compounds, provide a wide variety of colors, ranging from red-purple to blue-violet in diverse plant tissues, such as flowers, fruit, leaves, seeds, and roots [11–13]. Research into the biosynthetic pathways that are responsible for the synthesis of anthocyanins has been intense, and to date, much work has focused on the anthocyanin biosynthesis pathway (ABP) in plants [14, 15]. Cyanidin (Cy), delphinidin (Del), pelargonidin (Pel), peonidin (Peo), petunidin (Pet), and malvidin (Mal) comprise the core compounds that are involved with ABP, through three primary branches. Studies have revealed that deep red hues are frequently attributed to Cy, whereas orange-red and blue hues are typically ascribed to Pel and Del, respectively [16]. Many of the “structural” genes that encode the enzymes of the ABP have been cloned and characterized in different plants, including chalcone synthase (CHS), chalcone isomerase (CHI), flavanone 3-hydroxylase (F3H), dihydroflavonol 4-reductase (DFR), anthocyanin synthase (ANS), and so on [17–24]. At present, there are relatively few studies devoted to further elucidating the overall molecular mechanisms of anthocyanin synthesis in red maple.

The chlorophyll catabolic pathway leads to the accumulation of colorless breakdown products, which serve as one of the major sources of color change in leaves [25, 26]. The chlorophyll breakdown pathway consists of three common steps: chlorophyll-a synthesis, chlorophyll cycle, and chlorophyll degradation [27]. Genes for the common enzymes in this pathway include glumly-tRNA reductase (HEMA) [28–30], Mg-chelatase (CHLH/D/I) [29–33], chlorophyllase (CLH) [34], pheophorbide a oxygenase (PaO) [26, 27, 35–37], and red Chl catabolite reductase (RCCR) [38], which have been shown to be closely associated with the loss of visible color in a diverse range of plants [39–41]. Further, carotenoids are also considered by researchers to be an important class of pigments, due to their characteristic colors that range from yellow to red. Carotenoids always accumulate in plant chromoplasts to give them their distinctive hue. For example, several orange, yellow, or red foods such as corn, bananas, pumpkin, oranges, tomato, and watermelon obtain their color from carotenoids. The molecular mechanisms of the metabolic biosynthetic pathway for carotenoids in plants have been comprehensively reported. Recently, phytoene synthase (PSY) [42–45], phytoene desaturase (PDS) [46], ζ-carotene desaturase (ZDS) [47], lycopene β-cyclase (lcy B) [48], and lycopene ε-cyclase (lcy E) [49, 50] were found to have associations with carotenoid biosynthesis. However, the molecular mechanisms that regulate these three metabolic pathways in red maple have not been investigated as yet.

Currently, whole-transcriptome sequencing for non-model organisms, without reference sequences, has become an efficient tool for the extraction of useful genes and exploration of gene expression patterns. Third-generation sequencing technologies have been employed to generate highly contiguous reconstructions of many dozens of plants, such as *Agaves* [51], *Salvia miltiorrhiza* [52], rice [53], *Beta vulgaris* [54], *Triticum aestivum* [55], strawberry [56], sorghum [57], maize [58], *Amborella trichopoda* [59], *Phyllostachys edulis* [60], sugarcane [61], and *Arabidopsis* [62], which have provided new insights into their evolution and sequence diversity. PACBIO RS (Pacific Biosciences of California, Inc., http://www.pacificbiosciences.com/) created a platform for SMRT sequencing; hence, transcriptome sequencing technologies have been continually improved. Further, solutions toward addressing the higher raw error rate issue (10 to 15%) have been developed via several algorithmic techniques, including correction with NGS reads [63], or self-correction via circular-consensus (CCS) reads [64]. Thus, for this paper, we generated a complete *A. rubrum* transcriptome by combining both NGS and SMRT approaches. Through a combination of chemical analysis and bioinformatics, the anthocyanin, chlorophyll, and carotenoid metabolic pathways related to red maple leaf pigmentation were deduced, and candidate structural genes were examined. Therefore, this study established the foundation for further investigations into the mechanisms of leaf development in red maple.

## Results

### Pigment levels in green, red, and yellow leaves

Generally, maple leaves change in color from green to red or yellow in late autumn. HPLC and the alcohol extraction method were used to investigate these physiological changes. The content of six anthocyanins, chlorophyll, and carotenoids of different colored leaves were investigated.

**Fig. 1:**
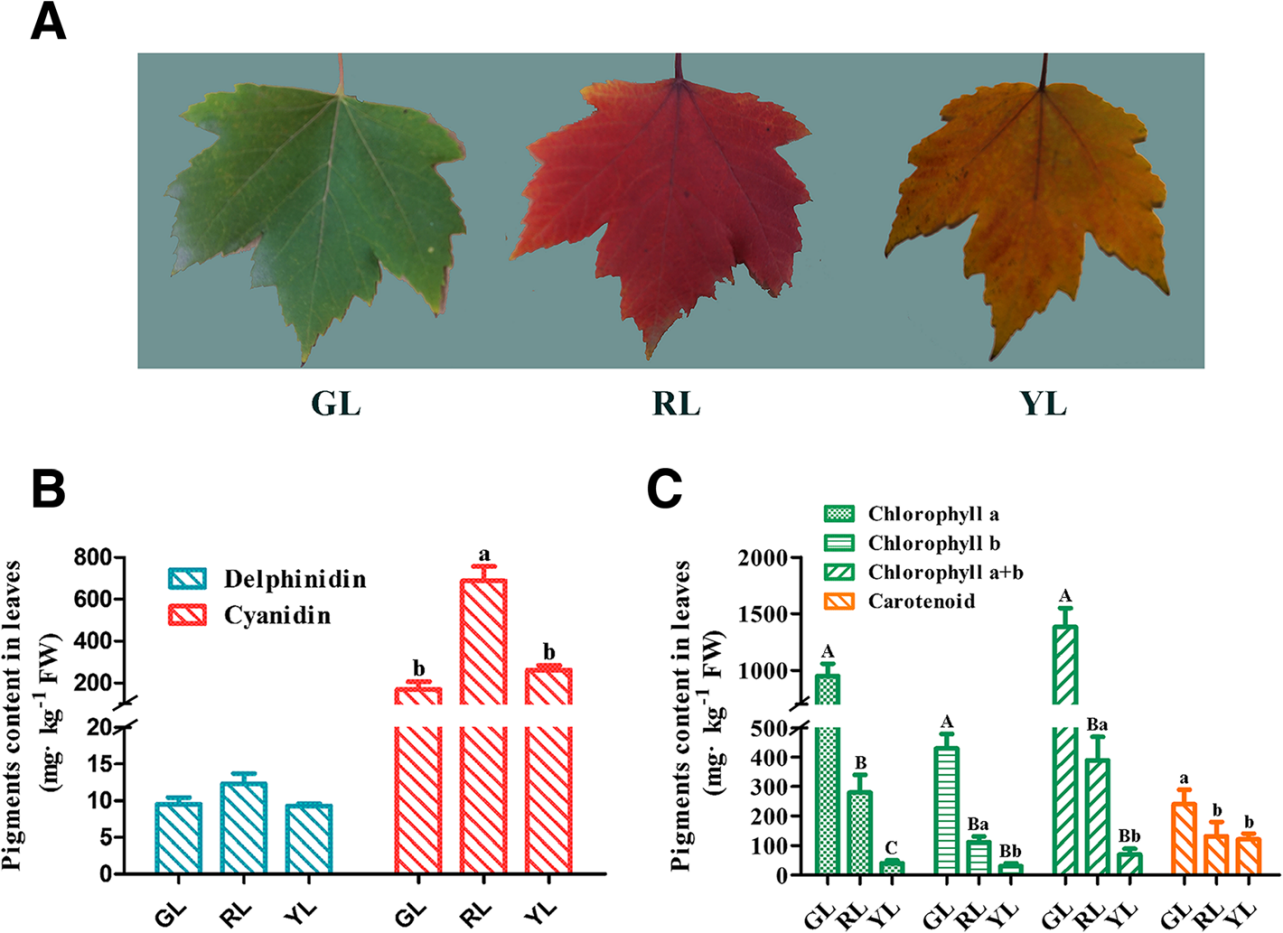
Morphology of red maple leaves and pigment accumulation in different colored leaves. **a** Different leaf colors of red maple. **b** Anthocyanin composition obtained by HPLC from green, red, and yellow leaves of red maple. **c** Chlorophyll-a, chlorophyll-b and carotenoid content of green, red, and yellow leaves of red maple. Error bars show standard error (SE) of the mean. GL, green leaves; RL, red leaves; YL, yellow leaves. Different lowercase letters above the error bars indicate significant difference of correlation at 0.05 level (One way ANOVA, *p*-value < 0.05); Different capital letters above the error bars indicate significant difference of correlation at 0.01 level (One way ANOVA, *p*-value < 0.01)

As can be seen from part B of Fig. 1, the anthocyanin constituents in the three types of leaves were consistent. Two anthocyanin compounds are responsible for red maple color pigmentation (Cy and Del, respectively), which is dominated by Cy. The accumulation of Cy in RL was three-fold higher than that of GL and YL. The quantity of Del in GL was comparable to RL and YL. In contrast, it was significant that we did not find Pel, Peo, Pet, or Mal in any of these leaves.

As shown in Fig. 1c, the chlorophyll content of the different colored leaves exhibited a decreasing tendency from GL, RL, to YL, and the variation trend of chlorophyll-b content was the same as the tendency reflected for chlorophyll-a. The content of chlorophyll-a collected in GL and RL was twice that of chlorophyll-b. The chlorophyll-a content of YL was lowest, and was no higher than chlorophyll-b. Further, the carotenoid content of GL was highest, with RL and YL following in succession. The carotenoid level in RL was similar to that detected in YL but was about half as much as in GL.

### Combined red maple sequencing approach

To elucidate the molecular basis of color changes in red maple leaves, two experiments were carried out, using either Illumina RNA-seq or the PacBio RS platform. First, nine mRNA samples (GL, RL, and YL; each in triplicate) were obtained separately using Illumina technology. A total of 387,763,976,150-nt paired-end raw reads were produced, and after filtering these raw reads, we obtained a total of 369,969,926 high-quality clean reads: 195,855,956 reads, 174,113,970 reads, and 154,354,082 reads were generated for the GL, RL, and YL samples, respectively (Table 1). Second, full-length cDNAs from 18 poly (A) RNA samples (root, branch, petiole, as well as GL, RL, and YL) were pooled for SMRT sequencing. In total, 10,732,280 raw reads (8.94 billion bases) were assembled with a mean length of 834 nt, and a N50 length of 1966 nt using the PacBio RS platform. Next, a CCS was generated from subread BAM files and 154,557 multipass consensus reads were obtained. In total, 266,010 full-length reads were obtained by detecting the poly (A), as well as 5′ and 3′ primer, sequences. For the following, we employed LoRDEC software to correct the high error rates of SMRT subreads by using the NGS reads as input data. Subsequent to filtering using CD-HIT, 125,448 non-redundant reads were reserved, and the number of genes from different transcript length intervals (< 0.5, 0.5–1, 1–2, 2–3, and > 3 kb) produced by SMRT sequencing were 21,014, 4965, 23,192, 18,135, and 58,142, respectively (Table 2).

**Table 1:** Summary of transcriptome sequencing data obtained using Illumina technology

Sample	Raw Reads	Clean reads	Clean bases	Error(%)	Q20(%)	Q30(%)	GC(%)
RL_1	57,244,376	55,687,302	8.35G	0.02	97.06	95.51	44.13
RL_2	62,164,748	60,464,996	9.07G	0.02	96.91	95.29	43.77
RL_3	59,025,616	57,961,672	8.69G	0.02	96.61	91.10	44.03
GL_1	62,802,636	59,895,360	8.98G	0.02	96.92	95.29	43.80
GL_2	73,263,300	67,980,298	10.2G	0.02	97.18	95.66	43.95
GL_2	73,263,300	67,980,298	10.2G	0.02	97.18	95.66	43.95
YL_1	62,657,656	60,344,520	9.05G	0.02	96.67	94.94	44.88
YL_2	51,589,246	49,960,742	7.49G	0.02	96.06	94.04	43.99
YL_3	45,022,194	44,048,820	6.61G	0.02	96.70	91.33	44.30

Note: *RL* Red Leaves, *GL* Green Leaves, *YL* Yellow Leaves

**Table 2:** Comparison of PACBIO read quality from subreads and corrected reads

Transcripts length interval	< 500 bp	500-1kbp	1 k-2kbp	2 k-3kbp	>3kbp	Total
Number of transcripts	24,231	6458	32,276	22,181	69,411	154,557
Number of Genes	21,014	4965	23,192	18,135	58,142	125,448

### Functional annotation and classification

**Fig. 2:**
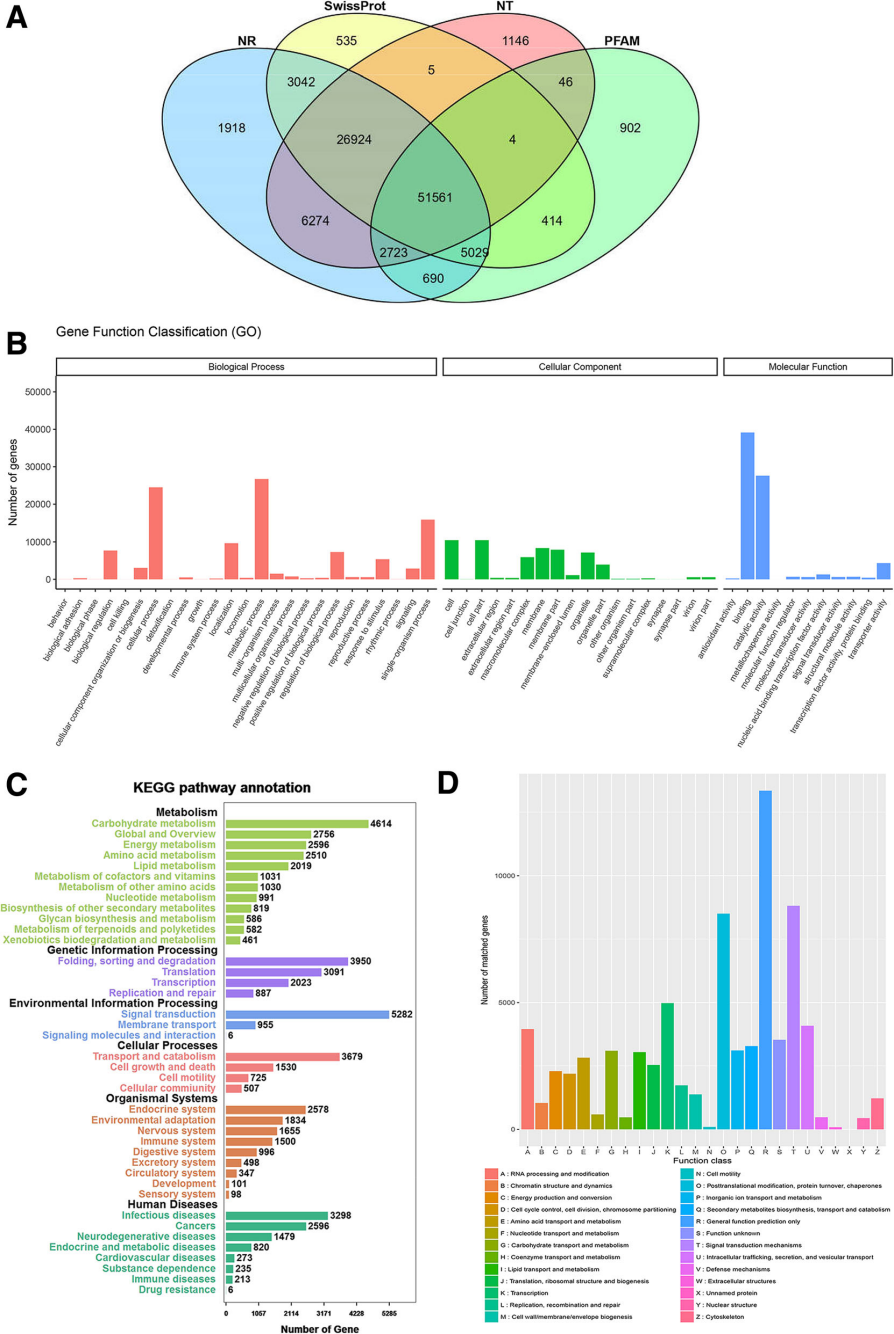
Functional annotation of unigenes in flower transcriptomes of *Acer rubrum* among different samples. **a** Venn diagram of number of transcripts annotated by BLASTx against protein databases. **b** GO classification of *Acer rubrum* transcripts. **c** KEGG pathway annotation of *Acer rubrum* transcripts. The vertical axis shows the annotations of the KEGG metabolic pathways. The horizontal axis represents the gene numbers annotated in each pathway. **d** COG classification of *Acer rubrum* transcripts. The capital letters on the horizontal axis indicate the COG categories, which are explained below the histogram, and those on the vertical axis indicate the number of genes

To achieve the putative functional annotation of the transcriptome, the assembled unigenes were examined against NR, NT, PFAM, and SWISS-PROT databases (Fig. 2). In total, 100,799 (80.35%) of the unigenes were annotated using at least one database; 51,560 (41.10%) of the unigenes could be assigned to a homolog in the abovementioned four databases. Among them, 98,161 (78.25%), and 88,683 (70.69%), could be annotated according to the NCBI, NR, and NT databases, respectively. In addition, 61,369 (48.92%), and 87,514 (69.76%) unigenes were annotated using the PFAM, and SWISS-PROT protein databases, respectively (Fig. 2a).

To further illustrate the primary biological functions of the red maple unigenes, GO, KOG, and KEGG pathway analyses were carried out. GO assignments described the predicted unigenes. A total of 61,370 unigenes were categorized into three gene ontology (GO) categories: cellular component (CC), biological process (BP), and molecular function (MF). These unigenes were further divided into 54 major functional groups. Of all the GO term categories, metabolic process (GO: 0008152), cell part (GO: 0044464), and binding (GO: 0005488) were the highest ranking in the three GO categories mentioned above, respectively. Cell killing (GO: 0001906), synapse (GO: 0045202) and metallochaperone activity (GO: 0016530) were the least frequent (Fig. 2b). A total of 97,539 unigenes were mapped to KEGG pathways, where carbon metabolism (ko01200, 1781 unigenes), spliceosome (ko03040, 1510 unigenes), and ubiquitin-mediated proteolysis (ko04120, 1426 unigenes) occupied the top three slots (Fig. 2c). Among the 125,448 unigenes, 69,181 (55.15%) unigenes were grouped into 26 KOG classifications. Group R (general function prediction only) was the most highly represented. Group T (signal transduction mechanisms) and O (post-translational modification, protein turnover, chaperones) also shared a high-percentage of genes. For unnamed proteins, extracellular structures, and cell motility groups, only a few genes were annotated for each (Fig. 2d).

### Identification of differentially expressed genes

**Fig. 3:**
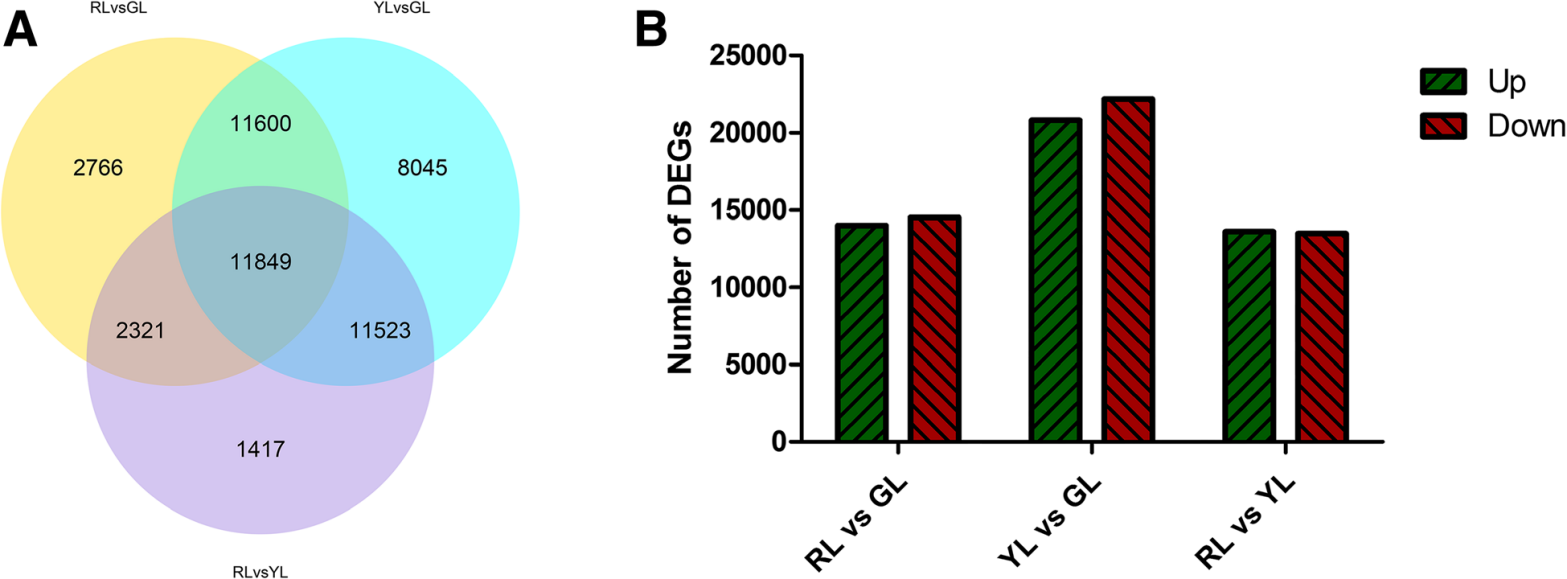
Statistics of differential expression genes (DEGs). **a** Venn diagram of differential expression genes. The sum of the numbers in each large circle represents the total number of differential expression genes in the comparison combination, whereas overlapping portions of the circles represent the differential expression genes shared between the combinations. **b** Statistical analysis of differential expression genes between trial groups

The Differentially Expressed Genes (DEGs) between different leaf colors were identified. A total of 49,521 DEGs were detected in at least one pairwise comparison (RL vs GL, YL vs GL, and RL vs YL) (Fig. 3a). In the comparison of YL vs GL, the number of DEGs (43,017) was much higher than the other two differences. Among them, 20,824 genes were up-regulated, and 22,193 genes were down-regulated. A comparison between the RL vs GL revealed that 28,536 were DEGs, with 14,009 up-regulated and 14,527 down-regulated unigenes. A total of 27,110 DEGs were identified in the RL vs YL comparison. Among them, 13,620 genes were up-regulated, and 13,490 genes were down-regulated (Fig. 3b).

GO functional enrichment analysis of DEGs revealed that of all the categories of GO terms between RL vs GL, catalytic activity (GO: 0003824) was the highest ranking in the three GO categories mentioned above, respectively, whereas virion membrane (GO: 0055036) was the least frequent. The main enrichment of the GO between YL vs GL and RL vs YL were all the metabolic process (GO: 0008152; Additional file 1: Figure S1). Further, the KEGG pathway enrichment analysis of DEGs was performed (Additional file 3: Table S2). Ribosome attained the top spot in the comparison of RL vs GL, YL vs GL. As an important secondary metabolite, the flavonoid biosynthesis pathway is an important metabolic pathway for plant pigment synthesis. In the comparison of RL vs YL, this pathway was significantly enriched.

### Comparison of transcriptional profiles of genes involved in anthocyanin biosynthesis

**Fig. 4:**
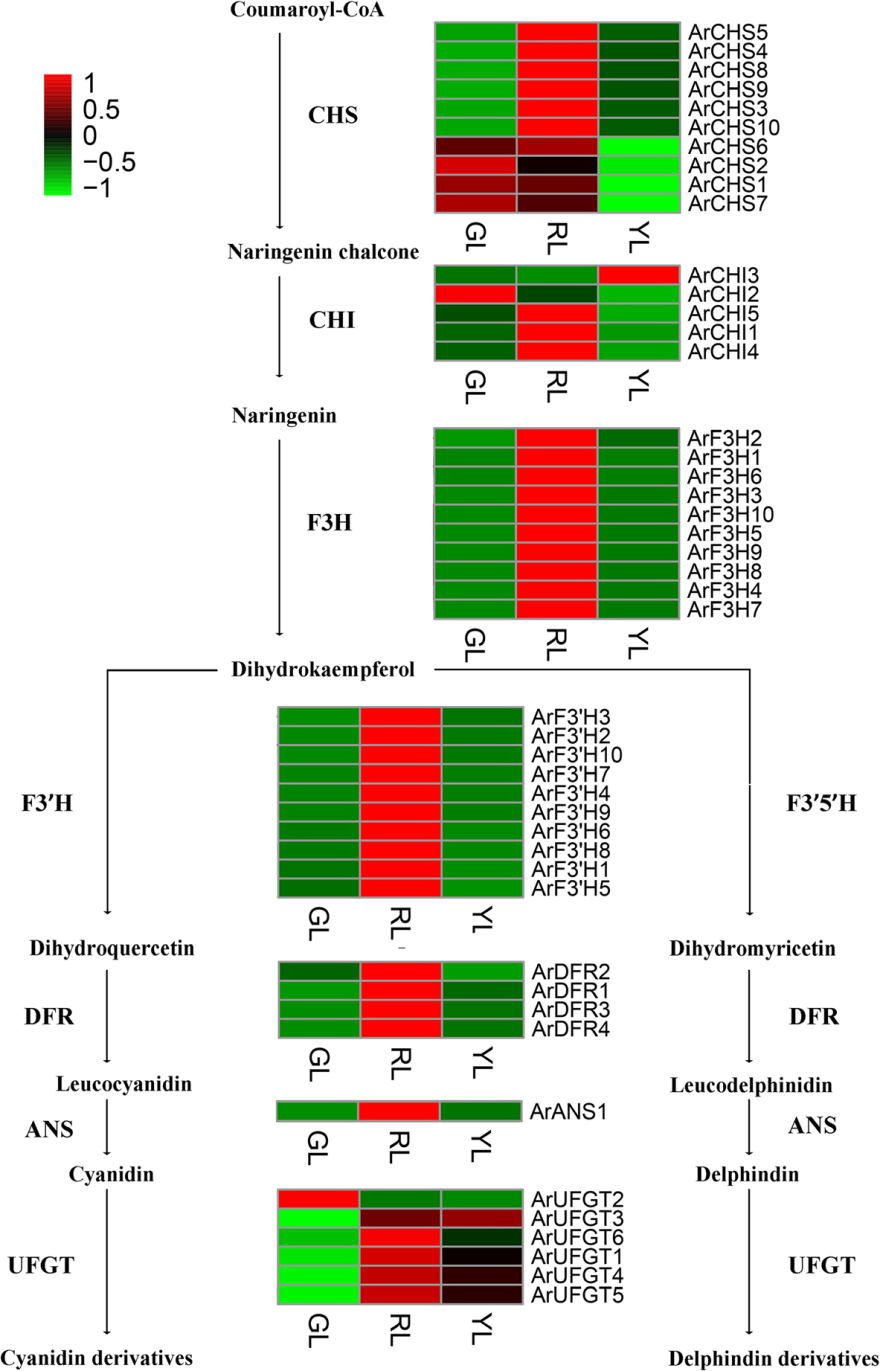
Heatmap of differentially expressed genes (DEGs) related to anthocyanin biosynthesis. GL, green leaves; RL, red leaves; YL, yellow leaves; CHS, chalcone synthase; CHI, chalcone isomerase; F3H, flavanone 3-hydroxylase; F3’H, flavanone 3′-hydroxylase; F3′5’H, flavanone 3’5’-hydroxylase; DFR, dihydroflavonol 4-reductase; ANS, anthocyanin synthase; UFGT, anthocyanidin 3-O-glucosyltransfersae

Previous research has demonstrated that the loss of anthocyanins directly influences the pigment content of leaves, and further affects the leaf expression of trees with color leaves. Accordingly, we investigated the enzyme-encoding genes that comprised the anthocyanin biosynthesis pathways (APBs). The analysis of our transcriptome data set revealed that 79 unigenes exerted a direct influence over eight enzymes that were known to be involved in anthocyanin biosynthesis, where 61 of these were identified as different expression genes. By removing extremely lowly expressed genes (FPKM< 1), 46 genes became the key candidates for discussion in our study (Additional file 4: Table S3). Aside from *ArANS*, which had one and only one member, the others (*ArCHS*, *ArCHI*, *ArF3H*, *ArF3’H*, *ArDFR*, *ArUFGT*, and *ArANR*) were demonstrated to be a multigene family. As shown in Fig. 4, all of these members were investigated in detail, and the results showed that the vast majority of the uni-transcripts were more highly expressed in RL, over GL, and YL. Approximately three quarters of these 46 core gene encoding enzymes involved in anthocyanin biosynthesis exhibited a similar expression pattern in that the total abundance of transcripts peaked in RL, were fewer in YL, and at a minimum in GL. Only a handful of different expression genes, including *ArCHI2*, *ArCHS1/2/6/7*, *ArFLS2*, and *ArUFGT2* exhibited higher expression in the GL over the other two leaf colors. Moreover, in YL, the *ArCHI3* and *ArUFGT3* expression levels were higher than that of GL and RL. This indicated good agreement between the changes in anthocyanin accumulation, and those in the expression of gene encoding pathway enzymes.

### Expression patterns of genes involved in chlorophyll metabolism

It has been suggested that the chlorophyll content level has significant variable effects on the physiological processes of plants, including growth and de-greening. Chlorophyll metabolism is catalyzed by a series of enzyme complexes, and this metabolic pathway consists of three steps: chlorophyll biosynthesis, chlorophyll cycle, and chlorophyll degradation. The disruption of any of these reactions may result in leaf color mutations. Accordingly, we conducted a careful investigation of the core genes that encoded the enzymes involved in this metabolic pathway.

**Fig. 5:**
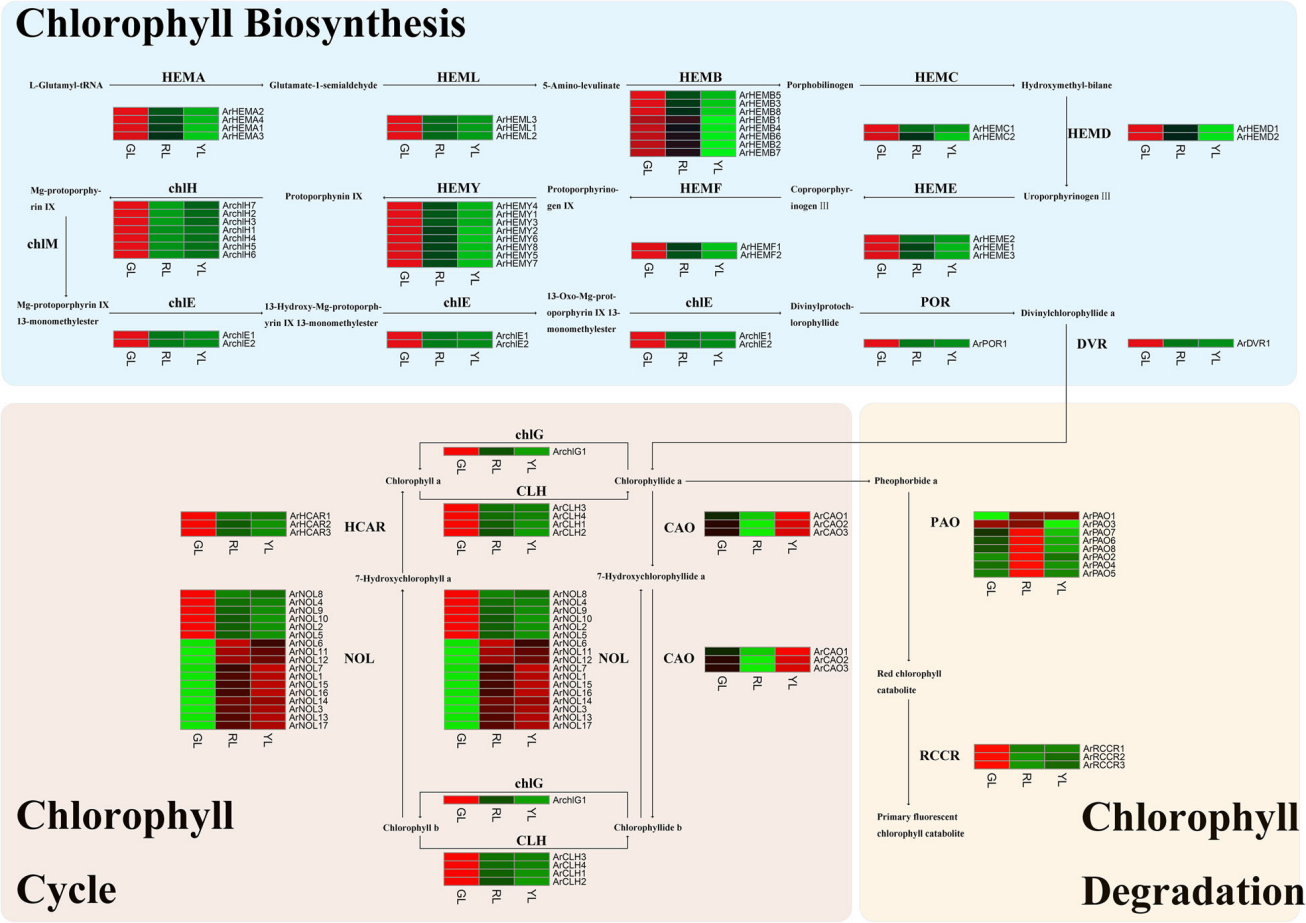
Heatmap of differentially expressed genes (DEGs) related to chlorophyll metabolism. GL, green leaves; RL, red leaves; YL, yellow leaves; HEMA, glumly-tRNA reductase; HEML, Glutamate-1-semialdehyde 2,1-aminomutase; HEMB, Glutamate-1-semialdehyde 2,1-aminomutase; HEMC, Glutamate-1-semialdehyde 2,1-aminomutase; HEMD, Uroporphyrinogen-III synthase; HEME, Uroporphyrinogen decarboxylase; HEMF, Coproporphyrinogen III oxidase; HEMY, Oxygen-dependent protoporphyrinogen oxidase; chlH, Magnesium chelatase subunit H; chlM, Magnesium-protoporphyrin IX methyltransferase; chlE, Magnesium-protoporphyrin IX methyltransferase; POR, protochlorophyllide reductase; DVR, divinyl chlorophyllide a 8-vinyl-reductase; CAO, chlorophyllide a oxygenase; chlG, chlorophyll synthase; CLH, chlorophyllase; HCAR, 7-hydroxymethyl chlorophyll-a reductase; NOL, non-yellow coloring-like; PaO, pheophorbide a oxygenase; RCCR, red chlorophyll catabolite reductase

Our results identified 146 candidate genes that encoded 20 enzymes related to chlorophyll metabolism and discovered 116 differential expression genes (DEGs). Following the removal of these genes with a FPKM value of less than 1, an intense focus was set on the remaining 88 genes in this study (Additional file 5: Table S4). Of these genes, three enzymes (*ArDVR*, *ArPOR*, and *ArchlG*) were found to belong to a single gene family, whereas the remaining genes belonged to multigene families. As depicted in Fig. 5, all of the DEGs related to chlorophyll biosynthesis were the most highly expressed in GL, where the expression patterns of most differential expression genes in this step were similar. The gene expression level of GL was the highest, followed by RL and YL. Beyond this, the *ArchlH* gene family was slightly different than the others, where the transcription level of these genes in YL was slightly higher than in RL. Earlier research results indicated that the transformation of chlorophyll-b to chlorophyll-a was the primary condition for the degradation of chlorophyll-b [65]. In our study, approximately half of the differential expressed genes associated with this process exhibited higher expression in GL.

The transcription levels of *ArNOL6*, *ArNOL11*, and *ArNOL12* were high in RL; primarily the latter two. Close to half of the *ArNOL* genes in YL were higher than in RL and GL. Moreover, all of the differential expression genes encoding PaO were involved in chlorophyll degradation, except for *ArPAO3* (i2_LQ_AR_c7492/f1p0/2494), which were more highly expressed in RL, which suggested that it was likely to play a key role in the further study of chlorophyll degradation.

### Expression patterns of genes involved in carotenoid metabolism

Carotenoids are one of the three largest classes of pigments, which have a significant influence on various biological processes in plants. They are also colorants that range from colorless to yellow, orange, and red, with variations reflected in many species. Previous studies confirmed that cis-lycopene, carotene, and xanthophylls are among the main carotenoid pigments in the photosystems of plants [66]. Therefore, we investigated the expression level of genes involved in this pathway.

**Fig. 6:**
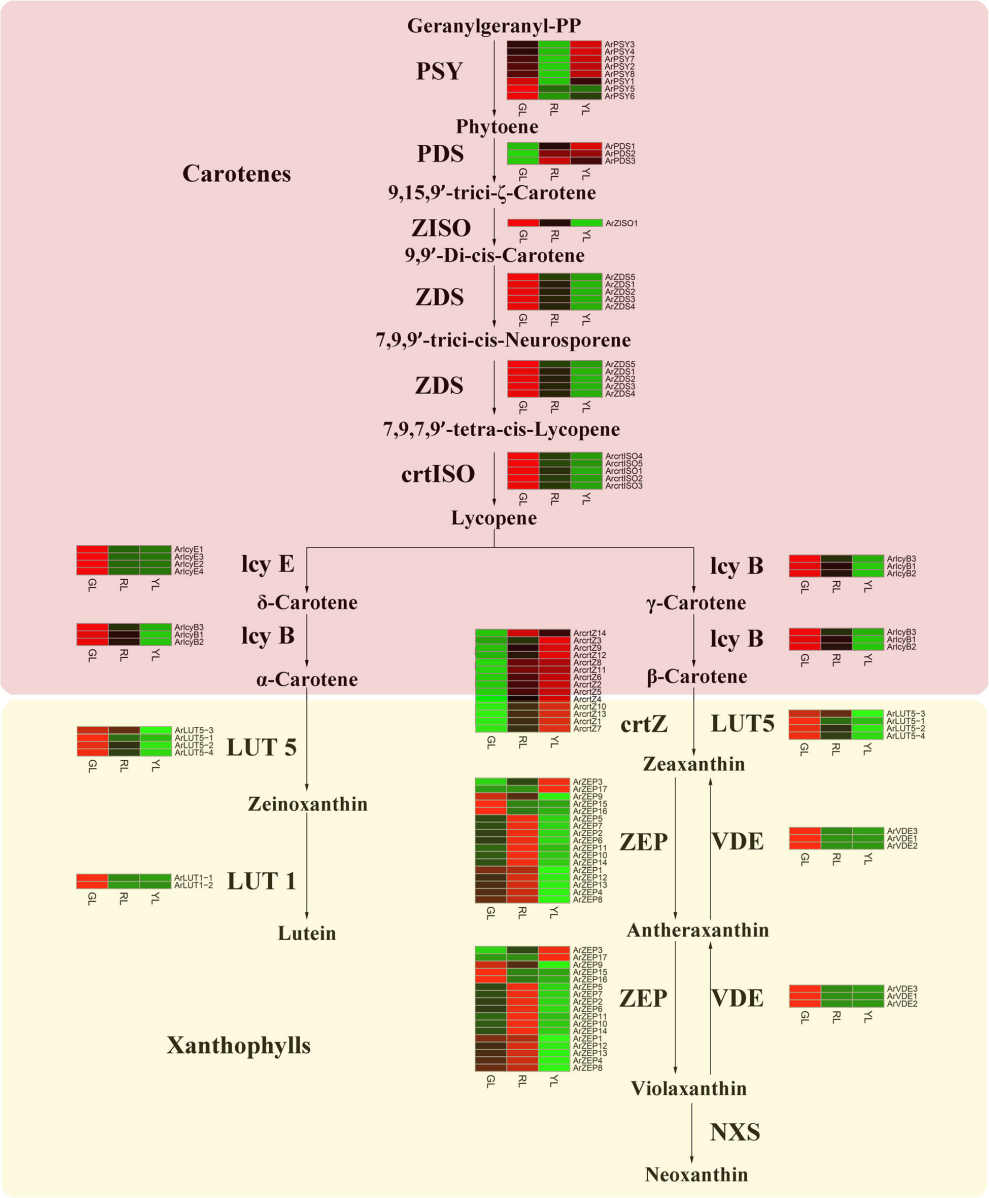
Heatmap of differentially expressed genes (DEGs) related to carotenoid biosynthesis. GL, green leaves; RL, red leaves; YL, yellow leaves; PSY, phytoene synthase; PDS, phytoene desaturase; ZISO, ζ-carotene isomerase; ZDS, ζ- carotene desaturase; CRTISO, carotenoid isomerase; lcyE, ε-cyclase; lcyB, β-cyclase; LUT5, β-hydroxylase; crtZ, β-carotene biosynthesis; LUT1, ε-cyclase; VDE, violaxanthin de-epoxidase; ZEP, zeaxanthin epoxidase; NXS, neoxanthin synthase

Our results revealed 114 candidate genes that encoded for 12 enzymes, which were related to carotenoid metabolism, and identified and 76 of these that were considered as DEGs. For this study, without those genes with a FPKM value of less than 1, our target moved to the remaining 69 genes (Additional file 6: Table S5). Aside from one enzyme (ArZISO1), which belonged to a single gene family, all of the others were demonstrated to be from multigene families. The first C40 carotenoid phytoene in the carotenoid pathway is derived through the condensation of two C20 GGPP via PSY molecules. As can be seen in Fig. 6, there were eight PSY genes identified in our study, with five of these genes being highly expressed in YL, while three had the highest expression in GL. In this reaction, the gene expression level of RL was the lowest. A previous study [67] found that the synthesis of lycopene had a direct relationship with four enzymes: PDS, z-carotene desaturase (ZDS), and two carotenoid isomerase (CRTISO, *Z-ISO*). Among these enzymes, there was no apparent regularity of PDS in the expression level of the three leaf types. Beyond this, the expression pattern of the three others were similar; the gene expression levels in GL were the highest, and with RL being higher than YL. In plants, lycopene cycling is a critical step in carotenoid metabolism, which generates diverse carotenoids, including δ-carotene, γ-carotene, α-carotene, and β-carotene. Two lycopene cyclases, *lcyE* and *lcyB*, are important for the determination of carotenoid content and composition. The results revealed that the observed *ArlcyE* and *ArlcyB* expression patterns were similar, and both *ArlcyE* and *ArlcyB* had the highest expression levels in GL, over RL and YL. The further hydroxylation of α-carotene and β-carotene produced xanthophylls (e.g., lutein and zeaxanthin) through *LUT1*, *LUT5*, *CrtR-b*, and *CrtZ*. These genes in our study demonstrated two distinct expression patterns, where the *ArLUT1* and *ArLUT5* showed higher expression in GL. Conversely, most differential expression genes encoding CrtR-b and CrtZ revealed that the transcription levels were high in YL. The ZEP and VDE enzymes are responsible for the processing of zeaxanthin to yield antheraxanthin and then violaxanthin. Most DEGs involved in this step exhibited higher expression in RL, than in GL and YL. Violaxanthin is converted to neoxanthin in the final step of the core carotenoid biosynthetic pathway, where neoxanthin synthase is the key enzyme in this process. No DEGs were found during this process.

## Discussion

Leaf pigments provide critical information as relates to the status of plant physiology. Chlorophylls play critical roles in photosynthetic reactions, while carotenoids and anthocyanins prevent damage to photosynthetic systems [68–70]. Pigment contents are very different and have varied compositions, such that the colors of plant leaves vary. In this study, we endeavored to elucidate the mechanism of color change involved in the differential pigmentation of red maples. The expression and metabolomic profiles of RL, YL, and GL were compared in terms of whole chlorophylls, carotenoids, and anthocyanin metabolism. Our findings suggested that the formation of RL and YL is the result of a combination of multiple pigments. Therefore, the abundance of the three metabolic-pathway candidate-genes was compared in these three colored leaves to uncover the secrets behind their changes in color.

**Fig. 7:**
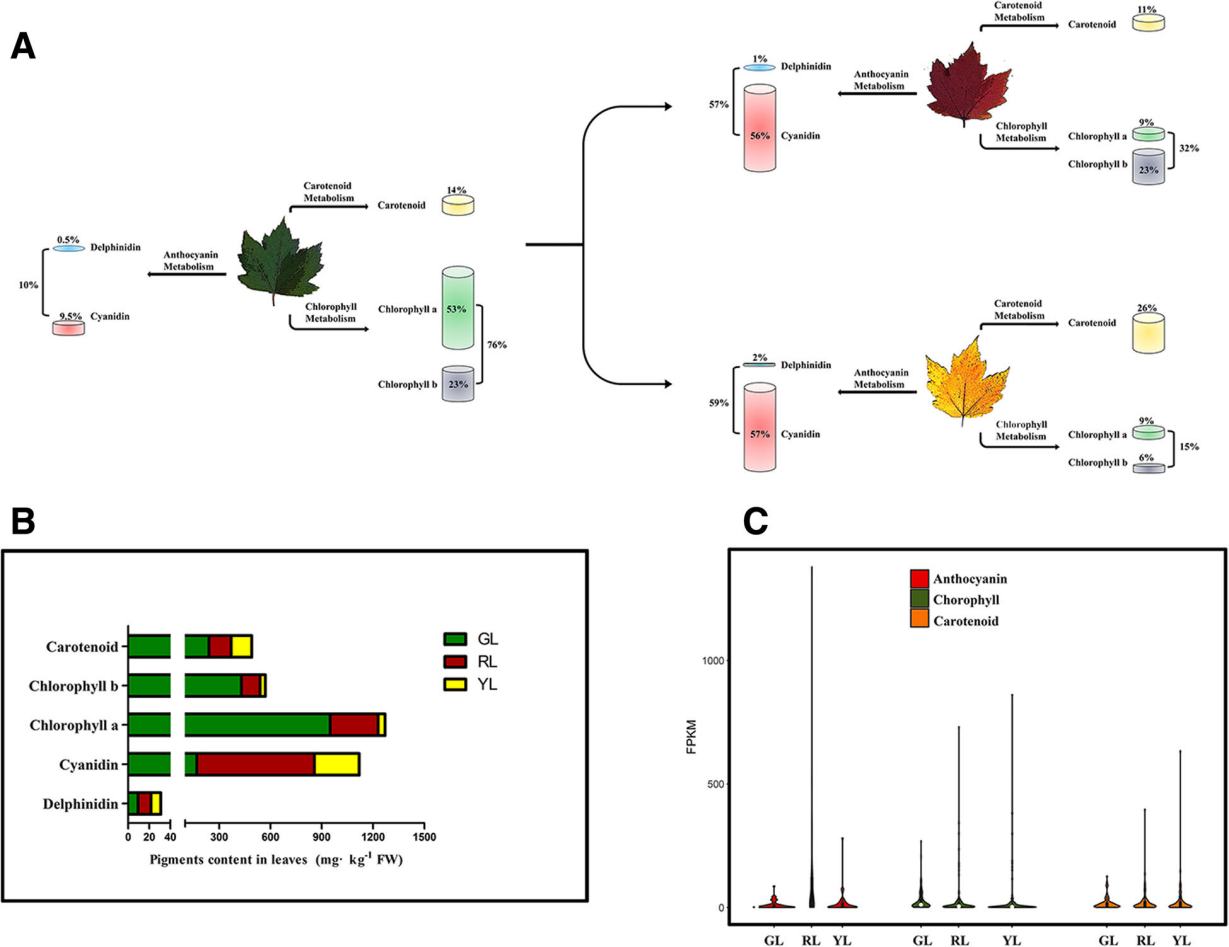
**a** Schematic diagram of the proportion of pigments in differently colored leaves. **b** Total pigment content in green, red, and yellow leaves. **c** The comparisons of the FPKM values of several pathways between green, red, and yellow leaves, expression of the violin. GL, green leaves; RL, red leaves; YL, yellow leaves

For a better description of the leaf pigment content, we considered the products of the three metabolic pathways in the leaves of each color as a whole and calculated the percentage of each pigment. As shown in Fig. 7a, Cy accounted for 9.5% of the total pigment in GL, compared with more than 56% in RL. The proportion of Cy totaled 57% in YL, although the content of Cy in YL was much less than that of RL (Fig. 7b). The relative expression level of the vast majority of unigenes involved in the Cy biosynthesis process in RL was the highest, followed by YL, with GL being the lowest (Fig. 7c). This result was consistent with many previous reports, that is to say, changes in Cy accumulation corresponded to alterations in the expression of the genes that encoded pathway enzymes [71–74]. Del levels were the lowest in GL, but close to those in RL and YL. Most Del and Cy-related reactions shared the same enzymes, as described above. The flavonoid 3′5’-hydroxylase (*F3’5’H*) determined the hydroxylation pattern of the B-ring, and was the key gene involved in the synthesis of Del [75]. Our results revealed that there was no significant difference in the gene expression of *F3’5’H*, and the expression of these genes in the colored leaves was extremely low, suggesting that *F3’5’H* was the most likely target for the inhibition of Del. The research of Boase et al. on cyclamen confirmed this speculation: flowers of the transgenic lines showed modified color (a shift in hue from purple to red/pink), which correlated positively with the loss of the endogenous *F3’5’H* transcript [16]. NakatSuka et al. also demonstrated that genetic engineering could reduce the expression of *F3’5’H* and related genes in gentian plants, which initiated modulations of flower color [76].

Our results showed that chlorophyll was the dominant factor that affected GL, which accounted for 76% of the total pigment in GL, far beyond 32% in RL and 15% in YL (Fig. 7a). In GL, chlorophyll-a accumulation was nearly three and a half-fold higher than in RL, and the content of chlorophyll-b in GL was close to four times higher than that in RL. YL contained only 4% chlorophyll-a and 7% chlorophyll-b content of GL (Fig. 7b). Considering that relatively low productivity in RL and YL still lagged behind that of GL, flux through chlorophyll metabolism in the former two would be limited. Chlorophyll metabolism is comprised of three steps: chlorophyll-a biosynthesis, chlorophyll cycling, and chlorophyll degradation [25, 77, 78]. Comparisons of the FPKM values of chlorophyll metabolism pathways between the three leaf colors is shown in Fig. 7c. Chlorophyll biosynthesis from glutamate to chlorophyll-a requires a 15-step reaction. Of all the unitranscripts involved in this process (49 genes engaging 12 enzymes) exhibited significantly up-regulated expression in GL. This explained why the content of chlorophyll-a in GL was much higher than that in RL and YL. The interconversion of chlorophyll-a and chlorophyll-b, referred to as the “chlorophyll cycle [79]”, is thought to be central in the adjustment of the chlorophyll a/b ratio under various physiological conditions. The chlorophyll a/b ratio was 2.2, 2.5, and 1.3 for GL, RL, and YL, respectively. It was reported that a portion of chlorophyll-a was converted to chlorophyll-b through the activity of Chlide a oxygenase (*CAO*). The overexpression of the *CAO* gene in *A. thaliana* might lead to a decrease in the chlorophyll a/b ratio in mature plants [80]. Chlorophyll-b can be reversibly converted to chlorophyll-a through 7-hydroxymethyl chlorophyll-a via chlorophyll-b reductase (*NOL/NYC*) and 7-hydroxymethyl chlorophyll-a reductase (*HCAR*). There are three members present in *A. rubrum*, and of these, all were found to be more highly expressed in YL, than in GL and RL. This might explain why the ratio of chlorophyll-a to chlorophyll-b in YL was the lowest. It has been widely reported in the literature that PAO encodes a Rieske-type monooxygenase, which explicitly catalyzes the oxidation of pheophytin a [81–86]. PAO activity provides the structural basis for all further breakdown products that are essential for the aging of leaves to yellow. Therefore, the chlorophyll degradation pathway is also called the ‘PAO pathway’. Our results indicated that the PAO expression level was higher in RL than that in GL and YL. These findings further explained why the content of chlorophyll-a in RL was lower than in GL.

For this study, the proportion of carotenoids in RL was 11%, which was lower than the proportion of the pigment in GL (14%). The proportion of carotenoids in the YL was 26%, which was higher than that of the GL (Fig. 7a). However, the carotenoid content of GL was 1.8 and 2.0 fold higher than RL and YL (Fig. 7b). The comparisons of the FPKM values of the carotenoid pathways between three color leaves are shown in Fig. 7c. Carotenoid pigments are primarily C40 lipophilic isoprenoids, and the quantity of accumulated carotenoids is determined by the rate of biosynthesis and degradation. The carotenoid biosynthetic pathway begins with the head-to-head condensation of two geranylgeranyl diphosphates by PSY to produce the initially colorless carotenoid 15-cis-phytoene. PSY is generally accepted as the rate-limiting step in the carotenoid biosynthetic pathway [87]. Our results revealed that highly expressed PSY genes were concentrated mainly in YL and GL, with relatively low expression levels in RL. While PSY is the major determinant for the carotenoid content of plants, other carotenoid biosynthetic enzymes alter accumulated carotenoids [88]. PDS, ZDS, and CRTISO play important roles in the generation of lycopene. *Lcy B* and *lcy E* were found to be primary regulators in the determination of the β-carotene/α-carotene ratio. Our results revealed that all of the above genes had the highest expression in GL, followed by RL and YL. At a downstream stage, the carotenoid biosynthetic pathway is hydroxylated to produce xanthophylls (e.g., lutein, zeaxanthin, and violaxanthin) by *LUT1*, *LUT5*, *CrtZ*, *ZEP*, and *VDE*. Lutein and zeaxanthin are positively correlated with the abundance of *LUT1*, *LUT5*, and *CrtZ* transcription. For the most part, these three genes exhibited higher expression in GL and YL. The accumulation of violaxanthin was associated with the expression of *ZEP* and *VDE*. Most of the DEGs involved throughout the synthesis of violaxanthin showed the highest expression in RL. The high levels of carotenoids in GL, in contrast to that of RL and YL, were consistent with the highest transcriptional levels of nearly all the carotenogenic genes at various stages of the carotenoid biosynthetic pathway. Our study suggested that PSY was the most likely target for carotenoid suppression in RL. The low levels of *PDS*, *ZDS*, *CRTISO*, *lcy B*, and *lcy E* might limit the flux through the carotenoid biosynthetic pathway in YL, while explaining the small quantities of carotenoids that accumulated in YL.

## Conclusions

In summary, we applied a combination of the SMRT platform and Illumina Hiseq technology to perform full-length transcript analysis of red maple. A comprehensive analysis of the coloration mechanism of red maple was conducted, which related the content levels of anthocyanins, chlorophyll, and carotenoids to the sequencing results of the transcriptome. Thus, the metabolism of major pigments in the generation of leaf color was elucidated, whereas the key regulatory genes that determined leaf color formation were analyzed and screened. Our results revealed that the leaves of *A. rubrum* are red due to the accumulation of anthocyanins, particularly the synthesis of Cy, which was higher in content and proportion than the other pigments. Yellow coloration was the result of the comprehensive effect of the decomposition of the chlorophyll pigments, the unmasking of carotenoid, pigments and the accumulation of Cy. Our research will assist with gaining further data as relates to the regulation of pigment anabolism, while providing valuable genetic resources for the improvement of leaf color.

## Methods

### Plant materials and sample preparation

For this study, a spontaneous leaf color mutant in the *A. rubrum* L. cultivar exhibited three leaf color phenotypes including green, yellow, and red from separate selected branches. The plant materials were planted in test plots at the Anhui Academy of Agricultural Sciences, Hefei, China (E 117.27, N 31.86). The roots, branches, petioles, as well as green leaves (GL), red leaves (RL), and yellow leaves (YL) were evenly harvested, respectively, for sequencing library constructions. For each leaf color, we considered 20 leaves as comprising one biological sample; each of the leaf colors collected were segregated as three independent biological replicates. All samples were flash frozen in liquid nitrogen, and stored at − 80 °C.

### Analysis of leaf pigment content

The frozen red maple leaves were finely ground to powder, whereafter 1 g was mixed into a 50 mL solvent mixture (the volume ratio of anhydrous alcohol - H_2_O - hydrochloric acid was 2:1:1) under ultrasonication for 30 min.. Prior to repeating the above steps, the extract was heated in a boiling water bath for 1 h, then rapidly cooled to room temperature. After stewing the filtrate for stratification, the supernatants were filtered via an ultrafiltration membrane. Subsequently, the leached solution was used for further analysis by high performance liquid chromatography (HPLC). Column type and conditions: column type, C18 column (250 mm * 4.6 mm, 5 μm); Mobile phase: mobile phase A (the volume ratio of HCOOH - H_2_O was 0.1: 100), mobile phase B (the volume ratio of HCOOH - CH_3_CN was 0.1: 100); Flow rate: 0.8 mL•min^− 1^. Gradient elution conditions are shown in Additional file 2: Table S1. Six authentic standards (Del, Cy, Pet, Pel, Peo, Mal) were obtained from Sigma-Aldrich China. The chlorophyll and carotenoid content were determined by an alcohol extraction method. Statistical significance was determined by one-way analysis of variance (ANOVA) with *p* < 0.05 considered as statistically significant. Analyses were carried out using SPSS 17.0 software (SPSS, Chicago, USA) and the GraphPad Prism 6.03 program was used to generate graphics.

### RNA quantification and qualification

Total RNA samples were isolated using the RNAprep Kit (DP441; Tiangen,). RNA purity was verified using a NanoPhotometer® spectrophotometer (IMPLEN, CA, USA). The Qubit® RNA Assay Kit in Qubit® 2.0 Fluorometer (Life Technologies, CA, USA) was used for RNA concentration. RNA degradation and contamination were monitored on 1% agarose gels. The total RNA was quantified, and the quality was assessed using the RNA Nano 6000 Assay Kit of the Bioanalyzer 2100 system (Agilent Technologies, CA, USA).

### Transcriptome sequencing library preparation

The Iso-Seq library was prepared as follows: (1) Oligo (dT) enriched mRNA; (2) Reverse transcription of mRNA into cDNA using the Clontech SMARTer PCR cDNA Synthesis Kit; (3) PCR amplification of the enriched cDNA; (4) Fragment screening using the BluePippin Size Selection System; (5) Full-length cDNA for damage repair, end repair, and connection of SMRT dumbbell joints; (6) Exonuclease digestion. The library was qualified and sequenced using the PacBio Sequel platform based on the effective concentration of the library and the data output requirements.

### Data processing

The Pact Bio software package SMRTlink was employed to process the original offline data. The circular consensus sequence (CCS) was generated from subread BAM files. Subsequently, according to whether the sequence contained a 5′ end, 3′ end, or the polyA tail, it was divided into a full-length, and non-full-length sequence. The full-length sequence was clustered by isoform-level clustering (ICE) to obtain a cluster consensus sequence, which was followed by final arrow polishing. The obtained consensus sequence was calibrated employing a non-full-length sequence to obtain a high-quality sequence, and subsequent analysis was performed.

### Error correction using Illumina reads

Additional nucleotide errors were corrected using Illumina RNAseq data with higher accuracy using LoRDEC [89]. The calibration process was divided into the following three steps: (1) build a DBG (de Bruijn Graph) graph using second-generation short reads; (2) read each of the three generations of long reads in turn, and judge whether there was second generation data support; (3) correct the data without second generation data and output the corrected sequence.

### Redundancy removal

Any redundant and similar sequences were removed by sequence alignment clustering using CD-HIT [90] software, and finally a non-redundant sequence file was output. The corrected transcript sequence was de-redundant according to a 99% similarity.

### Functional gene annotation

To obtain comprehensive information on gene function, we performed gene function annotations for seven databases, including: Nr (non-redundant protein sequences) [91]; Nt (non-redundant nucleotide sequences); Pfam (Protein family, http://pfam.xfam.org/); KOG/COG (Clusters of Orthologous Groups of proteins) [92]; Swiss-Prot (A manually annotated and reviewed protein sequence database) [93]; KEGG (Kyoto Encyclopedia of Genes and Genomes) [94, 95]; and GO (Gene Ontology) [96].

### CDS prediction

We used the fault-tolerant model of ANGEL software [97] for CDS prediction analysis [9], primarily through the use of machine learning algorithms, to maximize the codon usage frequency and protein structure information of the input sequence.

### Quantification of gene expression level

We employed RSEM software [98] to compare the results for each sample, and further obtained the number of read counts for each transcript in each sample. Next, FPKM conversion was performed to analyze the gene expression level. In RNA-seq technology, FPKM is the number of fragments per kilobase length from specific gene per million fragments, taking into account the depth of sequencing and the effect of gene length on the count of fragments, and is currently the most commonly used method for estimating gene expression levels [99].

### Differential expression and KEGG enrichment analysis

The input data of gene differential expression analysis is the read count data obtained from gene expression level analysis. The analysis is divided into three key parts: (1) First normalize the read count; (2) calculate the hypothesis test probability (*p*-value) according to the model; (3) finally, multiple hypothesis test calibration is performed to obtain the FDR value (error discovery rate). *P* values are calculated using the Benjamini and Hochberg’s methods, and the differential gene screening criteria is |log2(FoldChange)| > 0 & P value < 0.05. We employed KOBAS software to test the statistical enrichment of differential expression genes in the KEGG pathways.

## Additional files

Additional file 1: **Figure S1.** GO functional enrichment analysis of Differentially Expressed Genes (DEGs). GL, green leaves; RL, red leaves; YL, yellow leaves. (TIF 14385 kb)

Additional file 2: **Table S1.** Gradient elution conditions. (XLSX 9 kb)

Additional file 3: **Table S2.** Most enriched pathway terms of Differentially Expressed Genes (DEGs). GL, green leaves; RL, red leaves; YL, yellow leaves. (XLSX 13 kb)

Additional file 4: **Table S3.** Candidate unigenes involved in anthocyanin biosynthesis. GL, green leaves; RL, red leaves; YL, yellow leaves. (XLSX 13 kb)

Additional file 5: **Table S4.** Candidate unigenes involved in chlorophyll metabolism. GL, green leaves; RL, red leaves; YL, yellow leaves. (XLSX 12 kb)

Additional file 6: **Table S5.** Candidate unigenes involved in carotenoid metabolism. GL, green leaves; RL, red leaves; YL, yellow leaves. (XLSX 12 kb)

## Data Availability

The datasets generated during and analysed during the current study are not publicly available due to the consideration that the paper has not yet been published, the release time of the data (NCBI; SRA accession number PRJNA531583) has been delayed but are available from the corresponding author on reasonable request.

## Abbreviations

ABP: Anthocyanin biosynthesis pathway
ANS: Anthocyanin synthase
BP: Biological process
CAO: Chlorophyllide a oxygenase
CC: Cellular component
CCS: Circular-consensus
CHI: Chalcone isomerase
chlE: Magnesium-protoporphyrin IX methyltransferase
chlG: Chlorophyll synthase
chlH: Magnesium chelatase subunit H
chlM: Magnesium-protoporphyrin IX methyltransferase
CHS: Chalcone synthase
CLH: Chlorophyllase
crtISO: Carotenoid isomerase
crtZ: β-carotene biosynthesis
Cy: Cyanidin
DBG: de Bruijn Graph
DEGs: Differentially expressed genes
Del: Delphinidin
DFR: Dihydroflavonol 4-reductase
DVR: Divinyl chlorophyllide a 8-vinyl-reductase
F3H: Flavanone 3-hydroxylase
FDR: Error discovery rate
GL: Green leaves
GO: Gene ontology
HCAR: 7-hydroxymethyl chlorophyll-a reductase
HEMA: Glumly-tRNA reductase
HEMB: Glutamate-1-semialdehyde 2:1-aminomutase
HEMC: Glutamate-1-semialdehyde 2:1-aminomutase
HEMD: Uroporphyrinogen-III synthase
HEME: Uroporphyrinogen decarboxylase
HEMF: Coproporphyrinogen III oxidase
HEML: Glutamate-1-semialdehyde 2:1-aminomutase
HEMY: Oxygen-dependent protoporphyrinogen oxidase
HPLC: High performance liquid chromatography
ICE: Isoform-level clustering
KEGG: Kyoto Encyclopedia of Genes and Genomes
KOG/COG: Clusters of Orthologous Groups of proteins
lcyB: β-cyclase
lcyE: ε-cyclase
LUT1: ε-cyclase
LUT5: β-hydroxylase
Mal: Malvidin
MF: Molecular function
NGS: Next-generation sequencing
NOL: Non-yellow coloring-like
Nr: Non-redundant
Nt: Non-redundant
NXS: Neoxanthin synthase
PACBIO RS: Pacific Biosciences of California
PaO: Pheophorbide a oxygenase
PDS: Phytoene desaturase
Pel: Pelargonidin
Peo: Peonidin
Pet: Petunidin
Pfam: Protein family
POR: Protochlorophyllide reductase
PSY: Phytoene synthase
RCCR: Red chlorophyll catabolite reductase
RL: Red leaves
SE: Standard error
SMRT: Single-molecule real-time
VDE: Violaxanthin de-epoxidase
YL: Yellow leaves
ZDS: ζ- carotene desaturase
ZEP: zeaxanthin epoxidase
ZISO: ζ-carotene isomerase

## References

[1] Chinese Flora Editorial Board of the Chinese Academy of Sciences (2004). Flora of China.

[2] Takayama K, Sun BY, Stuessy TF (2013). Anagenetic speciation in Ullung Island, Korea: genetic diversity and structure in the island endemic species, Acer takesimense (Sapindaceae). J Plant Res.

[3] Bi W (2016). Traditional uses, phytochemistry, and pharmacology of the genus Acer (maple): A review. J Ethnopharmacol.

[4] Fossen T, Andersen ØM (1999). Cyanidin 3-(2″,3″-digalloylglucoside) from red leaves of Acer platanoides. Phytochemistry.

[5] Schmitzer V (2009). Correlation between chromaticity values and major anthocyanins in seven Acer palmatum Thunb. cultivars. Sci Hortic.

[6] Schmitzer V (2009). Phase change modifies anthocyanin synthesis in Acer palmatum Thunb. (Japanese maple) cultivars. Acta Physiol Plant.

[7] Ji SB (1992). Galloylcyanidin glycosides from Acer. Phytochemistry.

[8] Hyun KJ, Sanghyun L, Ju CE (2018). *Acer okamotoanum* protects SH-SY5Y neuronal cells against hydrogen peroxide-induced oxidative stress. Food Sci Biotechnol.

[9] Tsujimura M, Nakahama C (1965). Chemical components in the leaves of Acer aizuense. II, Maple tannin. Nippon Nogei Kagaku Kaishi.

[10] Navarro-Nunez L (2009). Thromboxane a2 receptor antagonism by flavonoids: Structure− activity relationships. J Agric Food Chem.

[11] Davies KM, Albert NW, Schwinn KE (2012). From landing lights to mimicry: the molecular regulation of flower coloration and mechanisms for pigmentation patterning. Funct Plant Biol.

[12] Zhao D, Tao J (2015). Recent advances on the development and regulation of flower color in ornamental plants. Front Plant Sci.

[13] Yoshikazu T, Filippa B, Steve C (2009). Recent Progress of Flower Color Modification by Biotechnology. Int J Mol Sci.

[14] Winkelshirley B (2001). Flavonoid biosynthesis. A colorful model for genetics, biochemistry, cell biology, and biotechnology. Plant Physiol.

[15] Grotewold E (2006). The genetics and biochemistry of floral pigments. Annu Rev Plant Biol.

[16] Boase MR (2010). Isolation and antisense suppression of flavonoid 3′, 5′-hydroxylase modifies flower pigments and color in cyclamen. BMC Plant Biol.

[17] Gu C (2015). Constitutive Activation of an Anthocyanin Regulatory Gene PcMYB10.6 Is Related to Red Coloration in Purple-Foliage Plum. Plos One.

[18] Sun W (2015). Molecular and Biochemical Analysis of Chalcone Synthase from Freesia hybrid in flavonoid biosynthetic pathway. Plos One.

[19] Tai D (2014). A Malus crabapple chalcone synthase gene, McCHS, regulates red petal color and flavonoid biosynthesis. Plos One.

[20] Nishihara M, Nakatsuka T, Yamamura S (2005). Flavonoid components and flower color change in transgenic tobacco plants by suppression of chalcone isomerase gene. Febs Lett.

[21] Itoh Y (2002). Excision of transposable elements from the chalcone isomerase and dihydroflavonol 4-reductase genes may contribute to the variegation of the yellow-flowered carnation (Dianthus caryophyllus). Plant Cell Physiol.

[22] Zuker A (2002). Modification of flower color and fragrance by antisense suppression of the flavanone 3-hydroxylase gene. Mol Breed.

[23] Wang H (2011). Expression and tissue and subcellular localization of anthocyanidin synthase (ANS) in grapevine. Protoplasma.

[24] Nakamura N (2006). RNAi suppression of the anthocyanidin synthase gene in Torenia hybrida yields white flowers with higher frequency and better stability than antisense and sense suppression. Plant Biotechnol.

[25] Hörtensteiner S (2013). Update on the biochemistry of chlorophyll breakdown. Plant Mol Biol.

[26] Kräutler B (2016). Breakdown of Chlorophyll in Higher Plants—Phyllobilins as Abundant, Yet Hardly Visible Signs of Ripening, Senescence, and Cell Death. Angew Chem Int Ed Engl.

[27] Schelbert S (2009). Pheophytin pheophorbide hydrolase (pheophytinase) is involved in chlorophyll breakdown during leaf senescence in Arabidopsis. Plant Cell.

[28] Wu Z (2007). A Chlorophyll-Deficient Rice Mutant with Impaired Chlorophyllide Esterification in Chlorophyll Biosynthesis. Plant Physiol.

[29] Niu Y (2014). Grey leaves in an alpine plant: a cryptic coloration to avoid attack?. New Phytol.

[30] Li Y (2015). Comprehensive transcriptome analysis discovers novel candidate genes related to leaf color in a Lagerstroemia indica yellow leaf mutant. Genes Genomics.

[31] Papenbrock J (2000). Role of magnesium chelatase activity in the early steps of the tetrapyrrole biosynthetic pathway. Plant Physiol.

[32] Mochizuki N (2001). Arabidopsis genomes uncoupled 5 (GUN5) mutant reveals the involvement of Mg-chelatase H subunit in plastid-to-nucleus signal transduction. Proc Natl Acad Sci U S A.

[33] Zhang H (2006). Rice Chlorina-1 and Chlorina-9 encode ChlD and ChlI subunits of Mg-chelatase, a key enzyme for chlorophyll synthesis and chloroplast development. Plant Mol Biol.

[34] Takamiya KI, Tsuchiya T, Ohta H (2000). Degradation pathway(s) of chlorophyll: what has gene cloning revealed?. Trends Plant Sci.

[35] Pružinská A (2003). Chlorophyll Breakdown: Pheophorbide a Oxygenase Is a Rieske-Type Iron-Sulfur Protein, Encoded by the Accelerated Cell Death 1 Gene. Proc Natl Acad Sci U S A.

[36] Pružinská A (2005). Chlorophyll Breakdown in Senescent Arabidopsis Leaves. Characterization of Chlorophyll Catabolites and of Chlorophyll Catabolic Enzymes Involved in the Degreening Reaction. Plant Physiol.

[37] Roca M (2004). Analysis of the chlorophyll catabolism pathway in leaves of an introgression senescence mutant of Lolium temulentum. Phytochemistry.

[38] Pružinská A (2007). In Vivo Participation of Red Chlorophyll Catabolite Reductase in Chlorophyll Breakdown. Plant Cell.

[39] Lai B (2015). Transcriptomic analysis of Litchi chinensis pericarp during maturation with a focus on chlorophyll degradation and flavonoid biosynthesis. BMC Genomics.

[40] Wen CH, Lin SS, Chu FH (2015). Transcriptome Analysis of a Subtropical Deciduous Tree: Autumn Leaf Senescence Gene Expression Profile of Formosan Gum. Plant Cell Physiol.

[41] Ohmiya A (2014). Identification of genes associated with chlorophyll accumulation in flower petals. Plos One.

[42] Cazzonelli CI, Pogson BJ (2010). Source to sink: regulation of carotenoid biosynthesis in plants. Trends Plant Sci.

[43] Toledoortiz G, Huq E, Rodríguezconcepción M (2010). Direct regulation of phytoene synthase gene expression and carotenoid biosynthesis by phytochrome-interacting factors. Proc Natl Acad Sci U S A.

[44] Rodrigo MJ, Marcos JF, Zacarías L (2004). Biochemical and Molecular Analysis of Carotenoid Biosynthesis in Flavedo of Orange (Citrus sinensis L.) during Fruit Development and Maturation. J Agric Food Chem.

[45] Gady ALF (2012). Induced point mutations in the phytoene synthase 1 gene cause differences in carotenoid content during tomato fruit ripening. Molecular Breeding.

[46] Gao H (2011). *Light effect on carotenoids production and expression of carotenogenesis genes in citrus callus of four genotypes*. Acta Physiologiae Plantarum.

[47] Zhu C (2002). *cDNA cloning and expression of carotenogenic genes during flower development in Gentiana lutea*. Plant Molecular Biology.

[48] Zhang JC (2013). Two Lycopene β-Cyclases Genes from Sweet Orange ( Citrus sinensis L. Osbeck) Encode Enzymes With Different Functional Efficiency During the Conversion of Lycopene-to-Provitamin A. J Integr Agric.

[49] Kato M (2007). Accumulation of Carotenoids and Expression of Carotenoid Biosynthetic Genes and Carotenoid Cleavage Dioxygenase Genes during Fruit Maturation in the Juice Sacs of 'Tamami,' 'Kiyomi' Tangor, and 'Wilking' Mandarin. Engei Gakkai Zasshi.

[50] Bai Y (2011). Flavonoid-related basic helix-loop-helix regulators, NtAn1a and NtAn1b, of tobacco have originated from two ancestors and are functionally active. Planta.

[51] Gross SM (2013). De novo transcriptome assembly of drought tolerant CAM plants, Agave deserti and Agave tequilana. Bmc Genomics.

[52] Xu Z (2015). Full-length transcriptome sequences and splice variants obtained by a combination of sequencing platforms applied to different root tissues of Salvia miltiorrhiza and tanshinone biosynthesis. Plant J.

[53] Lu T (2015). Transcriptome-wide investigation of circular RNAs in rice. RNA.

[54] Minoche AE (2015). Exploiting single-molecule transcript sequencing for eukaryotic gene prediction. Genome Biol.

[55] Dong L (2015). Single-molecule real-time transcript sequencing facilitates common wheat genome annotation and grain transcriptome research. Bmc Genomics.

[56] Li Y (2017). Global identification of alternative splicing via comparative analysis of SMRT- and Illumina-based RNA-seq in strawberry. Plant J.

[57] Abdel-Ghany SE (2016). *A survey of the sorghum transcriptome using single-molecule long reads*. Nat Commun.

[58] Wang B (2016). Unveiling the complexity of the maize transcriptome by single-molecule long-read sequencing. Nat Commun.

[59] Liu Xiaoxian, Mei Wenbin, Soltis Pamela S., Soltis Douglas E., Barbazuk W. Brad (2017). Detecting alternatively spliced transcript isoforms from single-molecule long-read sequences without a reference genome. Molecular Ecology Resources.

[60] Wang T (2017). Comprehensive profiling of rhizome-associated alternative splicing and alternative polyadenylation in moso bamboo (Phyllostachys edulis). Plant J Cell Mol Biol.

[61] Hoang NV (2017). A survey of the complex transcriptome from the highly polyploid sugarcane genome using full-length isoform sequencing and de novo assembly from short read sequencing. Bmc Genomics.

[62] Zhu FY (2017). Proteogenomic analysis reveals alternative splicing and translation as part of the abscisic acid response in Arabidopsis seedlings. Plant J.

[63] Au KF (2013). Characterization of the human ESC transcriptome by hybrid sequencing. Pnas.

[64] Li Q (2015). High-accuracy de novo assembly and SNP detection of chloroplast genomes using a SMRT circular consensus sequencing strategy. New Phytol.

[65] Meguro M (2011). Identification of the 7-hydroxymethyl chlorophyll a reductase of the chlorophyll cycle in Arabidopsis. Plant Cell.

[66] Isaacson T (2002). Cloning of tangerine from tomato reveals a carotenoid isomerase essential for the production of beta-carotene and xanthophylls in plants. Plant Cell.

[67] Nisar N (2015). *Carotenoid metabolism in plants*. Molecular Plant.

[68] Gitelson AA (2002). *Assessing Carotenoid Content in Plant Leaves with Reflectance Spectroscopy¶*. Photochem Photobiol.

[69] Merzlyak MN, Solovchenko AE, Gitelson AA (2003). Reflectance spectral features and non-destructive estimation of chlorophyll, carotenoid and anthocyanin content in apple fruit. Postharvest Biol Technol.

[70] Merzlyak MN (2005). Apple flavonols during fruit adaptation to solar radiation: spectral features and technique for non-destructive assessment. J Plant Physiol.

[71] Castellarin SD, Gaspero GD (2007). Transcriptional control of anthocyanin biosynthetic genes in extreme phenotypes for berry pigmentation of naturally occurring grapevines. BMC Plant Biology.

[72] Lin-Wang K, Bolitho K, Grafton K (2010). An R2R3 MYB transcription factor associated with regulation of the anthocyanin biosynthetic pathway in Rosaceae. BMC Plant Biol.

[73] Feng C (2012). Transcriptomic analysis of Chinese bayberry (Myrica rubra) fruit development and ripening using RNA-Seq. Bmc Genomics.

[74] Yuan S (2013). *The development of EST-SSR markers in Lilium regale and their cross-amplification in related species*. Euphytica.

[75] Ishiguro K, Taniguchi M, Tanaka Y (2012). *Functional analysis of Antirrhinum kelloggii flavonoid 3′-hydroxylase and flavonoid 3′,5′-hydroxylase genes; critical role in flower color and evolution in the genus Antirrhinum*. J Plant Res.

[76] Nakatsuka T (2010). Genetic engineering of novel flower color by suppression of anthocyanin modification genes in gentian. J Plant Physiol.

[77] Masuda T, Fujita Y (2008). *Regulation and evolution of chlorophyll metabolism*. Photochem Photobiol Sci.

[78] Eckhardt U, Grimm B, Hörtensteiner S (2004). Recent advances in chlorophyll biosynthesis and breakdown in higher plants. Plant Mol Biol.

[79] Rüdiger W (2002). *Biosynthesis of chlorophyll b and the chlorophyll cycle*. Photosynthesis Research.

[80] Tanaka R (2001). *Overexpression of chlorophyllide a oxygenase (CAO) enlarges the antenna size of photosystem II in Arabidopsis thaliana*. Plant J.

[81] Sakuraba Y (2012). *STAY-GREEN and Chlorophyll Catabolic Enzymes Interact at Light-Harvesting Complex II for Chlorophyll Detoxification during Leaf Senescence in Arabidopsis*. Plant Cell.

[82] Gray J (1997). *A Novel Suppressor of Cell Death in Plants Encoded by the Lls1 Gene of Maize*. Cell.

[83] Yang M (2004). *The Wound-Inducible Lls1 Gene from Maize is an Orthologue of the Arabidopsis Acd1 Gene, and the LLS1 Protein is Present in Non-Photosynthetic Tissues*. Plant Mol Biol.

[84] Tang Y (2011). *Knockdown of OsPAO and OsRCCR1 cause different plant death phenotypes in rice*. J Plant Physiol.

[85] Gomez-Lobato ME, Civello PM, Martínez GA (2012). Effects of ethylene, cytokinin and physical treatments on BoPaO gene expression of harvested broccoli. J Sci Food Agric.

[86] Ma N (2012). Cloning and Expression Analysis of Wheat Pheophorbide a Oxygenase Gene TaPaO. Plant Mol Biol Rep.

[87] Lopezjuez E, Pyke KA (2004). Plastids unleashed: their development and their integration in plant development. Int J Dev Biol.

[88] Farré (2011). Nutritious crops producing multiple carotenoids – a metabolic balancing act. Trends Plant Sci.

[89] Salmela L, Rivals E (2014). LoRDEC: accurate and efficient long read error correction. Bioinformatics.

[90] Fu L (2012). CD-HIT: accelerated for clustering the next-generation sequencing data. Bioinformatics.

[91] Li W, Jaroszewski L, Godzik A (2002). Tolerating some redundancy significantly speeds up clustering of large protein databases. Bioinformatics.

[92] Tatusov RL (2003). The COG database: an updated version includes eukaryotes. Bmc Bioinformatics.

[93] Bairoch A, Boeckmann B (2000). The SWISS-PROT protein sequence data bank, recent developments. Nucleic Acids Res.

[94] Kanehisa M (2004). The KEGG resource for deciphering the genome. Nucleic Acids Res.

[95] Kanehisa M (2006). From genomics to chemical genomics: new developments in KEGG. Nucleic Acids Res.

[96] Young Matthew D, Wakefield Matthew J, Smyth Gordon K, Oshlack Alicia (2010). Gene ontology analysis for RNA-seq: accounting for selection bias. Genome Biology.

[97] SHIMIZU K, ADACHI J, MURAOKA Y (2006). ANGLE: A SEQUENCING ERRORS RESISTANT PROGRAM FOR PREDICTING PROTEIN CODING REGIONS IN UNFINISHED CDNA. J Bioinform Comput Biol.

[98] Li B, Dewey CN (2011). RSEM: accurate transcript quantification from RNA-Seq data with or without a reference genome. Bmc Bioinformatics.

[99] Trapnell C (2010). Transcript assembly and quantification by RNA-Seq reveals unannotated transcripts and isoform switching during cell differentiation. Nature Biotechnology.

