# Supplementary figures and images for: Transcriptome analysis based on a combination of sequencing platforms provides insights into leaf pigmentation in Acer rubrum
Source: BMC Plant Biol. 2019 Jun 6;19:240. doi: 10.1186/s12870-019-1850-7 (PMC6555730; doi:10.1186/s12870-019-1850-7)

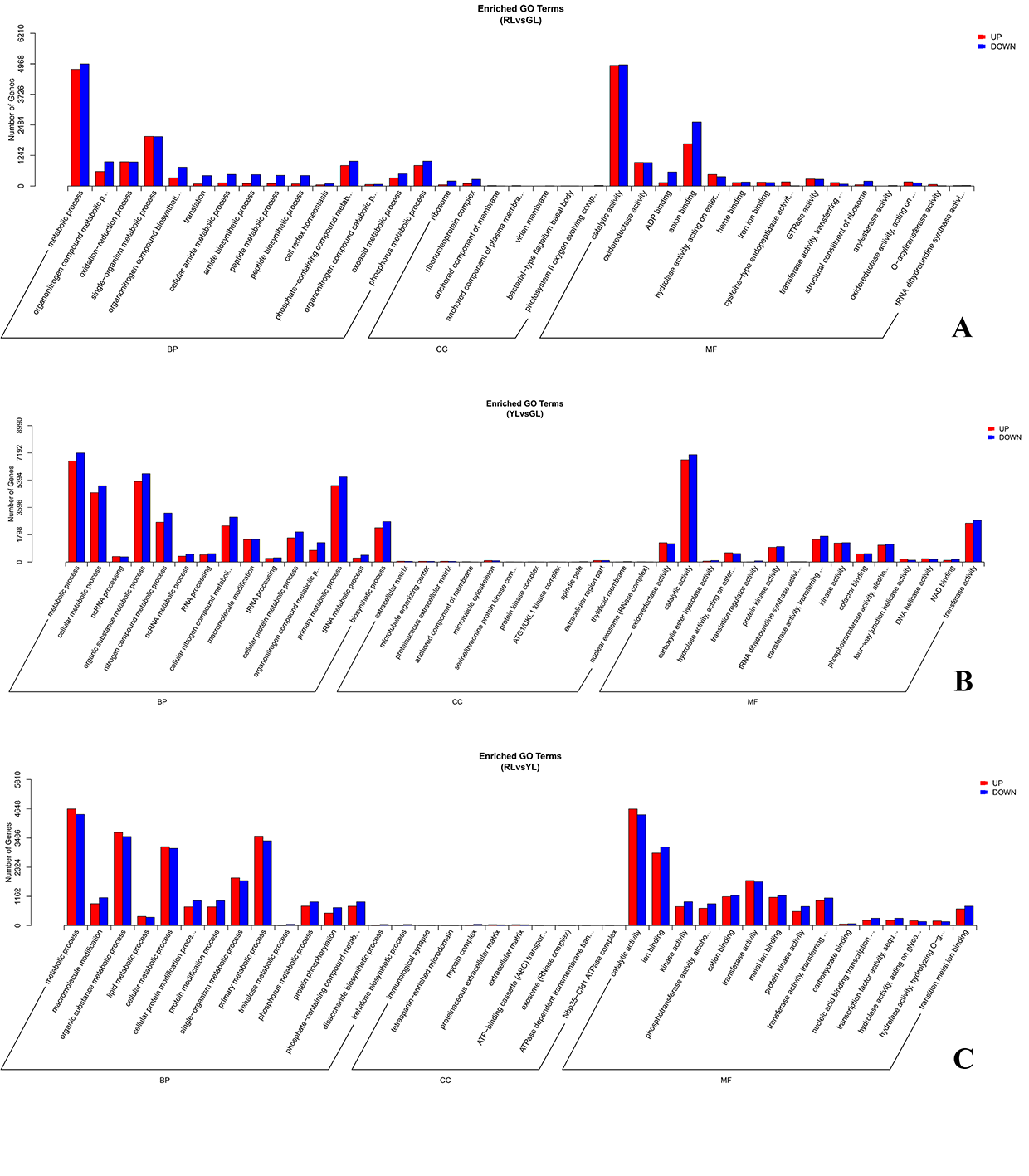

Supplement: Supplementary file 1 — Figure S1. GO functional enrichment analysis of Differentially Expressed Genes (DEGs). GL, green leaves; RL, red leaves; YL, yellow leaves. (TIF 14385 kb) [file 12870_2019_1850_MOESM1_ESM.tif]
